# 4-(Methylnitrosamino)-1-(3-Pyridyl)-1-Butanone Promotes Esophageal Squamous Cell Carcinoma Growth via Beta-Adrenoceptors *In Vitro* and *In Vivo*

**DOI:** 10.1371/journal.pone.0118845

**Published:** 2015-03-05

**Authors:** Nana Zhang, Xiujing Sun, Mingjiong Sun, Shengtao Zhu, Li Wang, Dan Ma, Yongjun Wang, Shutian Zhang, Peng Li

**Affiliations:** Department of Gastroenterology, Beijing Friendship Hospital, Capital Medical University, Beijing, China; Vanderbilt University, UNITED STATES

## Abstract

Cigarette smoke is a risk factor for esophageal squamous cell carcinoma (ESCC). It contains several carcinogens known to initiate and promote tumorigenesis as well as metastasis. The nitrosamine 4-(methylnitrosamino)-1-(3-pyridyl)-1-butanone (NNK) is one of the strongest carcinogens in tobacco and our previous studies have shown its proliferation-promoting role in the progression of ESCC. Recently, NNK was identified as an agonist for both beta1- and beta2-adrenoceptors. Thus, we hypothesized that the cancer-promoting effect of NNK was likely mediated through beta-adrenoceptors in ESCC. Therefore, we investigated the comprehensive role of NNK in ESCC *in vitro* and *in vivo*, and found that NNK promoted many oncogenic features including ESCC cell proliferation and xenograft tumor growth as well as ESCC cell migration and invasion. Western blotting showed that NNK induced significant up-regulation of phosphorylated ERK1/2, cyclin D1, Bcl-2, and vascular endothelial growth factor as well as down-regulation of Bax. Importantly, the oncogenic effects of NNK in ESCC and the altered protein expression were reversed to some extent by down-regulation of beta1- and beta2-adrenoceptors with the beta2-adrenoceptor showing a greater rescue effect. Taken together, our *in vitro* and *in vivo* results demonstrate that NNK plays an oncogenic role in ESCC through beta-adrenoceptors. Furthermore, beta2-adrenoceptor might play a more important role in this process. Our findings might provide a chemoprevention and therapy strategy for cigarette smoke-related ESCC carcinogenesis.

## Introduction

Esophageal cancer is one of the most frequently occurring malignancies worldwide with 481,000 new cases diagnosed each year [1]. Based on histology, more than 90% of esophageal cancers are categorized into either squamous cell carcinoma or adenocarcinoma. Esophageal squamous cell carcinoma (ESCC) accounts for most cases [2]. However, the prognosis of patients with ESCC remains unsatisfactory despite recent improvements in its diagnosis and treatment [3]. Thus, a clearer understanding of the tumorigenesis is urgently needed to improve early diagnosis and targeted therapies of ESCC.

The relationship between cigarette smoking and esophageal cancers has been proven by epidemiological studies [4,5]. Cigarette smoking is a major risk factor for both ESCC and adenocarcinoma of the esophagus. Ingestion of tobacco carcinogens, particularly nitrosamines, is thought to be the predominant risk factor for esophageal cancers [6,7]. The nitrosamine 4-(methylnitrosamino)-1-(3-pyridyl)-1-butanone (NNK) is one of the strongest carcinogens among nitrosamines and studies have shown its carcinogenetic action in the progression of pancreatic and lung cancers [8–10]. However, there are few studies investigating the role of NNK in ESCC. Our preliminary research revealed a proliferation-promoting effect of NNK in the progression of ESCC (unpublished data), but the molecular mechanism of this process is unclear.

Recently, NNK was identified as an agonist for both beta1- and beta2-adrenoceptors [11]. Several reports have shown that NNK stimulates the secretion of epinephrine and norepinephrine in cancer cells, which enhances tobacco-driven tumor development [12,13]. Further study found that the stimulatory effect of NNK on colon cancer cell proliferation can be inhibited by beta-blockers [14]. Consistent with these results, our previous study has shown that epinephrine promotes ESCC cell proliferation in a dose- and time-dependent manner, and a beta-adrenoceptor antagonist inhibits this process [15].

Thus, we hypothesized that beta-adrenoceptors might play a role in the prosurvival effect of NNK in ESCC. In line with this hypothesis, our previous study found that extracts from cigarette smoke (containing a number of toxic chemicals including NNK) stimulates the proliferation of ESCC cells and up-regulates the expression of beta-adrenoceptors. More importantly, this stimulatory effect on cell proliferation can be abolished by selective antagonists of beta1- and beta2-adrenoceptors [16]. In the present study, we aimed to identify the comprehensive function of NNK in the carcinogenesis of ESCC and the role of beta-adrenoceptors in this process *in vitro* and *in vivo*, and found that NNK plays an oncogenic effect in ESCC through beta-adrenoceptors.

## Results

### KYSE410 and HET-1A cells express both beta1- and beta2-adrenoceptors, and siRNA effectively inhibits their expression

**Fig 1:**
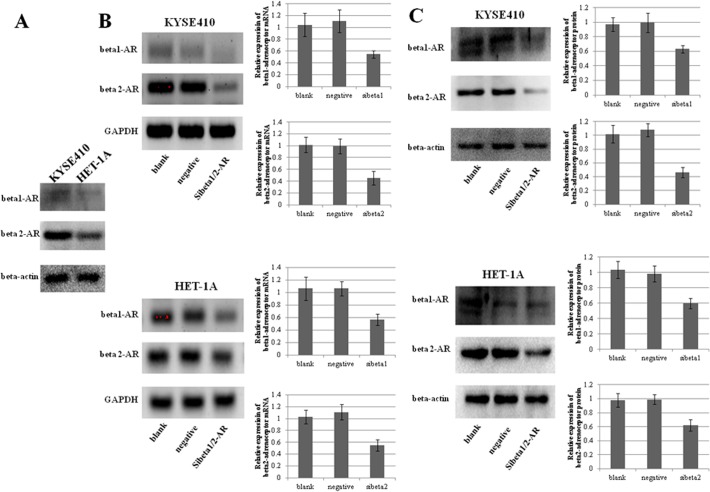
Expression of beta1- and beta2-adrenoceptors. A, Western blotting showed the expression of beta1- and beta2-adrenoceptors in KYSE410 cells, an ESCC cell line, and HET-1A cells, an immortalized esophageal epithelial cell line. B and C, siRNA-induced down-regulation of beta1- and beta2-adrenoceptor mRNA and protein expression was confirmed by RT-PCR (B) and western blotting (C), respectively. GAPDH and beta-actin served as endogenous controls. Negative is representative of nonspecific control siRNA for beta-adrenoceptors.

As beta-adrenoceptors have been shown to promote cancer cell growth [17], the expression of beta1- and beta2-adrenoceptors was determined by western blotting of KYSE410 cells, an ESCC cell line, and HET-1A cells, an immortalized esophageal epithelial cell line. Both KYSE410 and HET-1A cells expressed beta1- and beta2-adrenoceptors, and their expression levels were higher in KYSE410 cells (Fig. 1A).

To explore the role of beta-adrenoceptors in ESCC, we used small interfering RNA (siRNA) to down-regulate the expression of beta1- and beta2- adrenoceptors. Using RT-PCR and western blot analyses, we confirmed substantial reductions of the expression levels of beta1- and beta2-adrenoceptors in these cell lines by siRNA-mediated knockdown. The efficiency of the inhibition was 45%∼55% for mRNA expression, and 46%∼63% for protein expression (Fig. 1B and 1C).

Moreover, we include a second ESCC cell line, KYSE70 cells to confirm our data. Similar to KYSE410 and HET-1A cells, the expression levels of beta1- and beta2-adrenoceptors of KYSE70 cells were effectively knocked down by siRNA (S1 Fig.).

### NNK increases KYSE410 and HET-1A cell proliferation, which is reversed by RNA interference of beta1- and beta2-adrenoceptors

To study the effect of NNK and beta-adrenoceptors on KYSE410 and HET-1A cells, we performed 3-(4, 5-dimethyl-thiazol-2yl)-2, 5-diphenyltetrazolium bromide (MTT) assays to measure cell proliferation in variously treated groups (see Materials and methods) [18].

**Fig 2:**
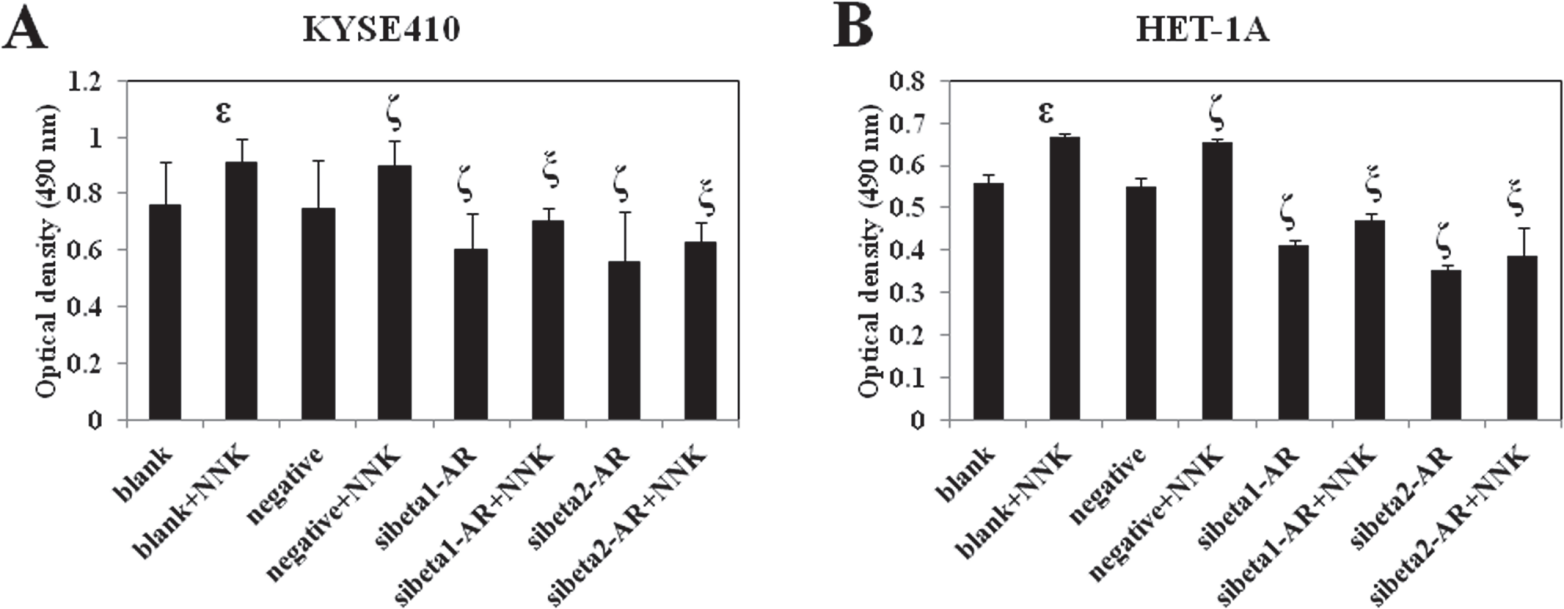
Effect of NNK and beta-adrenoceptors on cell proliferation. Variously treated KYSE410 (A) and HET-1A (B) cells were seeded in triplicate into 96-well plates. At the indicated time point, the medium was removed, MTT and DMSO were added sequentially, and the ODs were measured using a microplate reader. Data were obtained from three independent experiments. ^ε^ p<0.05 compared with the blank group; ^ζ^ p<0.05 compared with the negative group; ^ξ^ p<0.05 compared with the negative+NNK group. Negative is representative of nonspecific control siRNA for beta-adrenoceptors.

The optical density (OD) at 490 nm of each group is shown in Fig. 2A and 2B. NNK significantly promoted the proliferation of KYSE410 and HET-1A cells. Knockdown of beta1- and beta2-adrenoceptors inhibited the proliferation of KYSE410 and HET-1A cells. Moreover, down-regulation of both beta1- and beta2-adrenoceptors before treatment with NNK reversed the NNK-induced cell proliferation. Beta2-adrenoceptor showed a more prominent effect on blocking the NNK-enhanced cell proliferation.

To verify our results, we study the action of NNK and beta-adrenoceptors on KYSE70 cells. As shown in S2 Fig., NNK facilitated the growth of KYSE70 cells and knockdown of beta1- and beta2-adrenoceptors restrained the growth. Down-regulation of both beta1- and beta2-adrenoceptors before treatment with NNK reversed the NNK-induced cell proliferation. Beta2- adrenoceptors exhibited a more remarkable effect on blocking the NNK-enhanced cell proliferation.

The above results indicated that the promoting effect of NNK on cell proliferation can be reversed by RNA interference of both beta1- and beta2-adrenoceptors. In addition, the rescue effect of RNA interference of beta2-adrenoceptor was higher than that of beta1-adrenoceptor.

### NNK decreases KYSE410 and HET-1A cell apoptosis, which is reversed by RNA interference of beta1- and beta2-adrenoceptors

It has been reported that NNK inhibits apoptosis of lung and pancreatic cancer cells [9,19]. Thus, we hypothesized that NNK may have a suppressive effect on the apoptosis of esophageal epithelial cells and ESCC cells.

**Fig 3:**
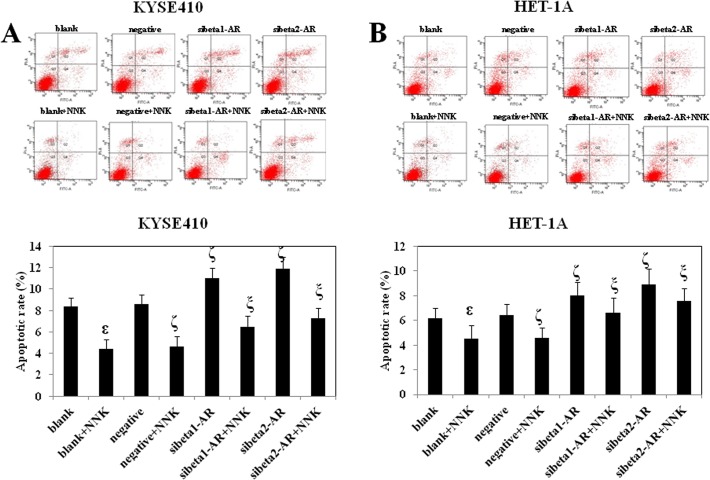
Effect of NNK and beta-adrenoceptors on cell apoptosis. Variously treated KYSE410 (A) and HET-1A (B) cells were stained with annexin V-FITC and PI, and then analyzed by flow cytometer. Annexin V-FITC is a sensitive probe that identifies apoptotic cells. PI is a standard viability probe for flow cytometry, which is used to distinguish viable from nonviable cells. Cells that stain positive for both annexin V-FITC and PI are either late apoptotic or necrotic cells (Q2). Early apoptotic cells stain positive for annexin V-FITC and negative for PI (Q4). Data are representative of three independent experiments. ^ε^ p<0.05 compared with the blank group; ^ζ^ p<0.05 compared with the negative group; ^ξ^ p<0.05 compared with the negative+NNK group. Negative is representative of nonspecific control siRNA for beta-adrenoceptors.

We performed an annexin V-FITC and propidium iodide (PI) assay to detect the effect of NNK and beta-adrenoceptors on the apoptosis of KYSE410 and HET-1A cells. The apoptosis rate of each cell type is shown in Fig. 3A and 3B. NNK significantly inhibited the apoptosis of KYSE410 and HET-1A cells. Knockdown of beta1- and beta2-adrenoceptors increased the apoptosis of KYSE410 and HET-1A cells. Furthermore, down-regulation of both beta1- and beta2-adrenoceptors before treatment with NNK reversed the inhibitive effect of NNK on cell apoptosis. Beta2- adrenoceptors had a greater effect on rescuing the NNK-inhibited cell apoptosis. The above results indicated that the effect of NNK on inhibition of cell proliferation can be reversed by RNA interference of both beta1- and beta2-adrenoceptors. In addition, RNA interference of beta2-adrenoceptor reversed the effect of NNK more significantly than that of beta1-adrenoceptor.

### NNK promotes KYSE410 cell migration and invasion, which is reversed by RNA interference of beta1- and beta2-adrenoceptors

ESCC is one of the most aggressive cancers, and its metastasis is a critical determinant of a poor prognosis. Thus, it is important to explore the effect of NNK and beta-adrenoceptors on the migration and invasion of ESCC cells. In this study, we used two types of modified Boyden chamber to investigate the migration and invasion of KYSE410 cells [20].

**Fig 4:**
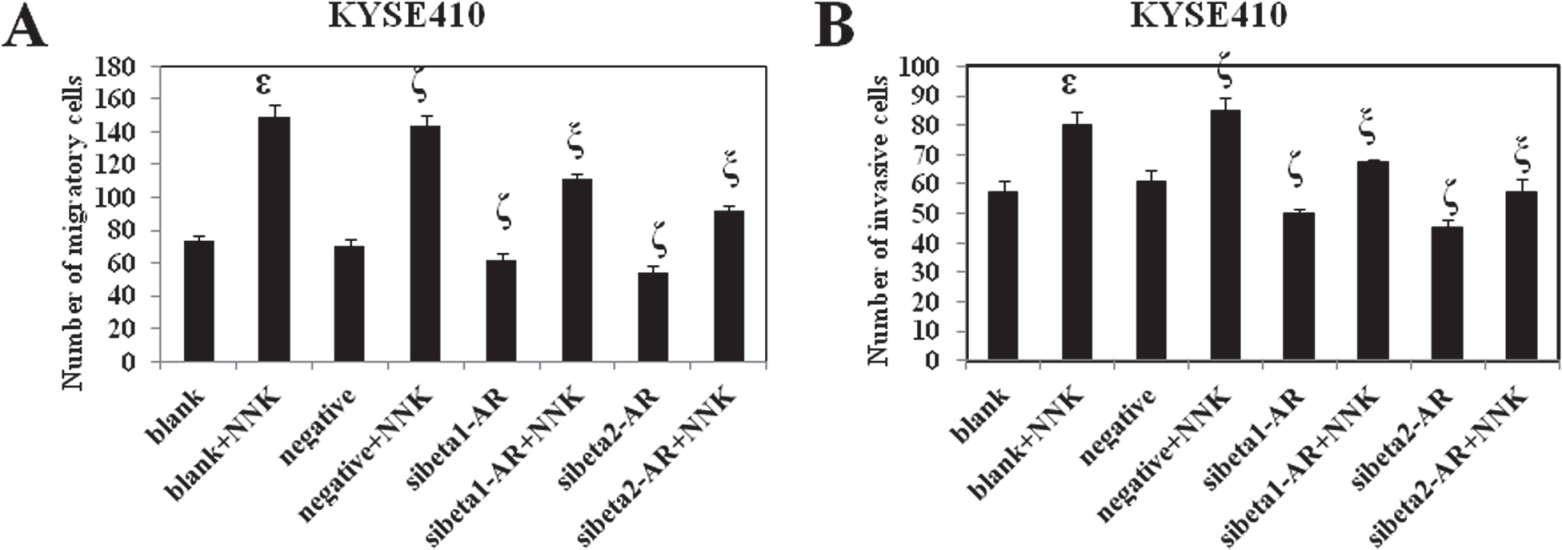
Effect of NNK and beta-adrenoceptors on cell migration and invasion. Variously treated KYSE410 cells were seeded in Boyden chambers for migration or invasion assays. At the end of the experiments, migratory or invasive cells that were on the bottom surface were fixed, stained with hematoxylin and eosin, and then counted under a microscope at 200×. Data were obtained from three independent experiments. ^ε^ p<0.05 compared with the blank group; ^ζ^ p<0.05 compared with the negative group; ^ξ^ p<0.05 compared with the negative+NNK group. Negative is representative of nonspecific control siRNA for beta-adrenoceptors.

First, we used a chamber containing a polyethylene terephthalate membrane filter to examine the migration of KYSE410 cells. The number of migratory cells in each group is shown in Fig. 4A. NNK significantly promoted the migration of KYSE410 cells. Knockdown of beta1- and beta2-adrenoceptors inhibited the migration of KYSE410 cells. Moreover, down-regulation of both beta1- and beta2-adrenoceptors before treatment with NNK reversed NNK-enhanced cell migration. Beta2-adrenoceptors showed a more striking effect on cancelling the NNK-promoted cell migration.

Compared with migration, invasion is more closely related to the process of metastasis because invasive cells need to breakdown the extracellular matrix before transversing membranes. Thus, we used a modified Boyden chamber containing a polyethylene terephthalate membrane coated with matrigel to investigate the invasion of KYSE410 cells. The number of invasive cells is shown in Fig. 4B. NNK prominently facilitated the invasion of KYSE410 cells. Knockdown of beta1- and beta2-adrenoceptors inhibited the invasion of KYSE410 cells. Furthermore, down-regulation of both beta1- and beta2-adrenoceptors before treatment with NNK reversed the NNK-enhanced cell invasion. Beta2- adrenoceptors exhibited a more remarkable effect on blocking the NNK-promoted cell invasion.

The above results indicated that the effect of NNK on promotion of cell migration and invasion can be reversed by RNA interference of both beta1- and beta2-adrenoceptors. In addition, RNA interference of beta2-adrenoceptor reversed the effect of NNK more significantly than that of beta1-adrenoceptor.

### Effects of treatment with NNK and RNA interference of beta-adrenoceptors on p-ERK1/2, cyclin D1, Bcl-2, VEGF, and Bax expression

**Fig 5:**
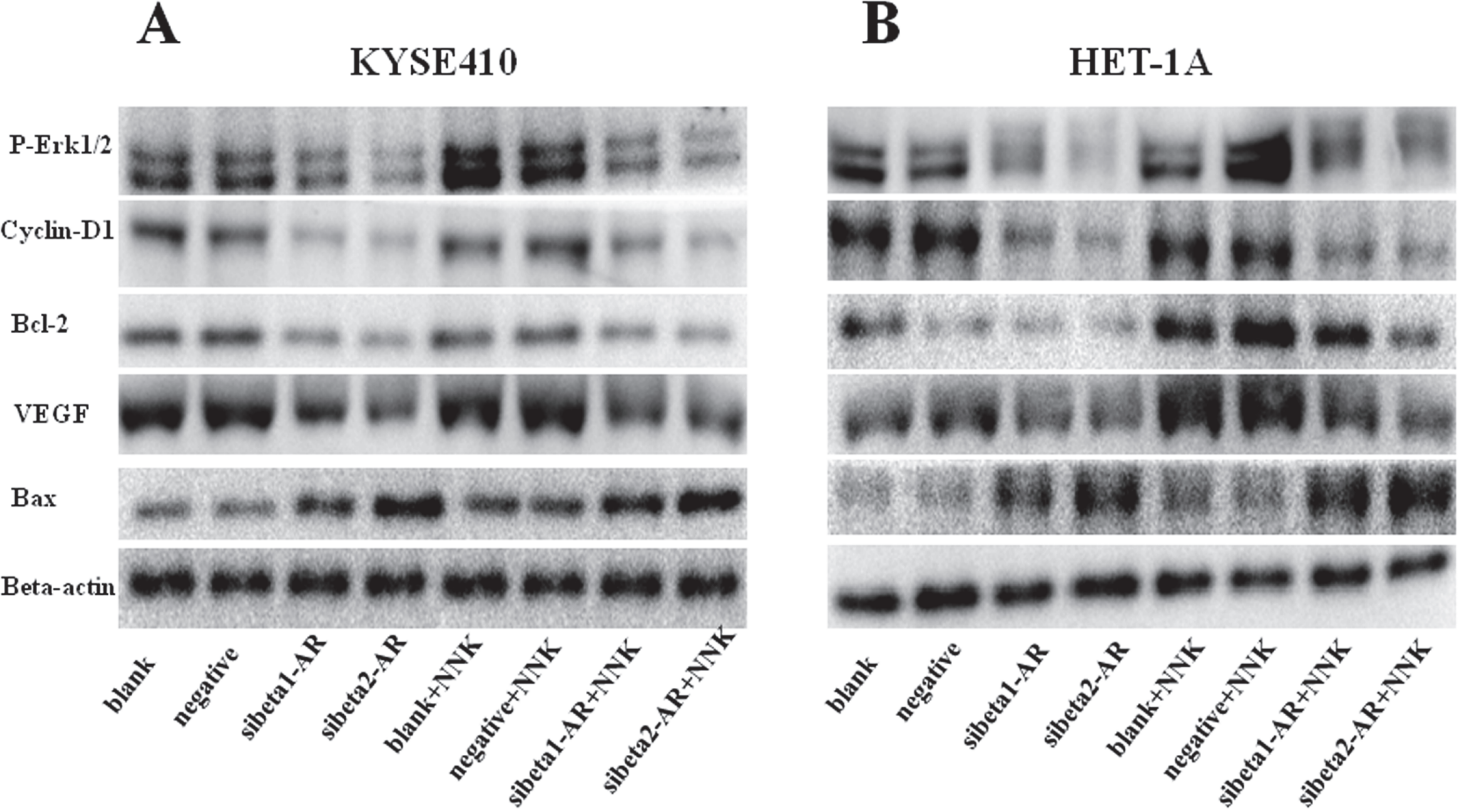
Effect of NNK and beta-adrenoceptors on the expression p-ERK1/2, cyclin-D1, Bcl-2, VEGF, and Bax. NNK promoted the expression of p-ERK1/2, cyclin-D1, Bcl-2, and VEGF, and decreased the expression of Bax in KYSE410 (A) and HET-1A (B) cells. Knockdown of beta-adrenoceptors decreased the expression of p-ERK1/2, cyclin D1, Bcl-2, and VEGF, and increased the expression of Bax. More importantly, down-regulation of both beta1- and beta2-adrenoceptors before treatment with NNK reversed the NNK-associated protein expression, and beta2-adrenoceptors showed a more significant effect. Beta-actin served as an endogenous control. Independent experiments were repeated three times. Negative is representative of nonspecific control siRNA for beta-adrenoceptors.

We detected the expression of several proteins that are critical for driving a number of cancer processes, including cell survival, proliferation, and motility, by western blotting. The results are shown in Fig. 5. NNK increased the expression of p-ERK1/2, cyclin D1, Bcl-2, and VEGF, and decreased the expression of Bax. Knockdown of beta-adrenoceptors decreased the expression of p-ERK1/2, cyclin D1, Bcl-2, and VEGF, and increased the expression of Bax. Moreover, the effect of beta2-adrenoceptor was more significant than that of beta1-adrenoceptor. Importantly, down-regulation of both beta1- and beta2-adrenoceptors before treatment with NNK reversed the NNK-associated protein expression, and beta2- adrenoceptor showed a more significant effect.

### Effect of NNK, atenolol, and ICI118551 on the tumorigenicity of ESCC cells *in vivo*

Our above experiments indicated that NNK and beta-adrenoceptors played important roles in ESCC cell proliferation, apoptosis, migration, and invasion *in vitro*. Therefore, we performed animal experiments to further investigate the function of NNK and beta-adrenoceptors in the pathogenesis of ESCC *in vivo*. We used atenolol (a selective antagonist of beta1-adrenoceptor) and ICI118551 (a selective antagonist of beta2-adrenoceptor) to explore the effect of beta-adrenoceptors.

**Fig 6:**
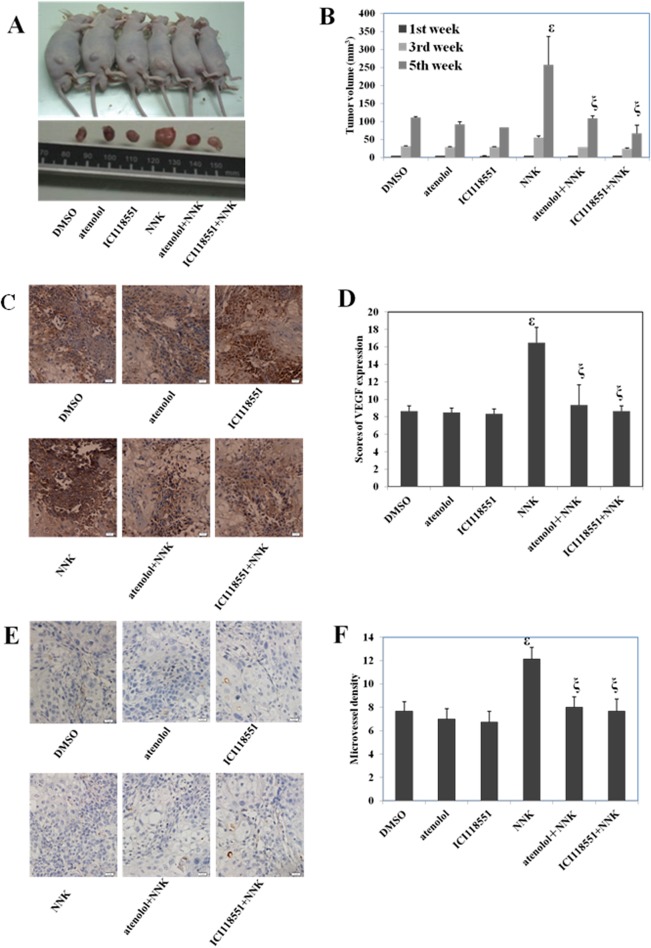
Effect of NNK, atenolol, and ICI118551 on the tumorigenicity of ESCC cells *in* vivo. KYSE410 cells were subcutaneously injected into the right posterior flank of nude mice. At 1 week post-implantation, the mice were randomized into six groups with six animals in each group to receive various treatments: DMSO, atenolol (10 mg/kg/day), ICI118551 (10 mg/kg/day), NNK (1.2 mg/kg/day), atenolol+NNK, and ICI118551+NNK. **A,** Representative images of the subcutaneous tumors formed in nude mice. **B,** Histograms showing the average tumor volume ± SD per group. **C,** VEGF expression in tumors. **D**, Histological scores of VEGF expression in each group. **E,** CD31 expression in tumors. **F,** Microvessel density (MVD) of each group. Data are the means ± SD. ^ε^ p<0.05 compared with the blank group; ^ξ^ p<0.05 compared with the NNK group.

Nude mice were divided into six variously treated groups (see Materials and methods). At 1 week post-implantation, tumors had developed in every group. As time progressed, tumors in the NNK group grew faster than those in any other group (Fig. 6A and Table 1). The average tumor volume of the NNK group was significantly larger than that of the control group. However, there was no significant difference between atenolol/ICI118551 and control groups. Importantly, both atenolol+NNK and ICI118551+NNK groups formed significantly smaller tumors than those in the NNK group (Fig. 6B). Furthermore, there was no significant difference between atenolol+NNK and ICI118551+NNK groups. Thus, the results of the *in vivo* experiments were consistent with those of our *in vitro* experiments. NNK increased ESCC cell oncogenicity, which was reversed by blockade of beta1- and beta2-adrenoceptors.

**Table 1:** Tumor volumes in nude mice after treatment with NNK and beta-adrenoceptor antagonists.

	**Tumor volume (mm^3^, mean±SD)**		
Group	1^st^ week	3^rd^ week	5^th^ week
DMSO	4.59±0.25	31.48±0.66	111.43±2.44
atenolol	4.71±0.06	29.51±0.77	91.92±6.89
ICI118551	4.55±0.13	29.16±0.74	83.41±0.69
NNK	4.59±0.20	55.41±4.60	257.79±77.91^ε^
atenolol+NNK	4.69±0.15	28.76±0.22	108.49±6.80^ξ^
ICI118551+NNK	4.71±0.06	24.41±1.79	67.20±22.17^ξ^

^ε^ p<0.05 compared with the blank group

^ξ^ p<0.05 compared with the NNK group.

Because angiogenesis is essential for tumor growth and metastasis, we detected the VEGF and CD31 expression of xenografts in nude mice by immunohistochemistry to further explore the role of NNK and beta-adrenoceptors *in vivo*. VEGF is the most studied pro-angiogenic molecule in tumor angiogenesis. CD31 is a vascular endothelial cell surface marker and widely used to assess microvessel density (MVD). The results are shown in Fig. 6C–6F. The NNK group had significantly higher expression of VEGF and a higher MVD than those in the control group. There was no significant difference between atenolol/ICI118551 and control groups. Importantly, both atenolol+NNK and ICI118551+NNK groups had significantly lower expression of VEGF and a lower MVD than those in the NNK group. No significant difference in VEGF expression and MVD was found between atenolol+NNK and ICI118551+NNK groups.

## Discussion

It is well known that cigarette smoking is an epidemiological risk factor for ESCC [2,5]. However, which components in tobacco and which molecular mechanisms are involved in this process are still unclear. NNK is a tobacco-specific nitrosamine that has a high affinity for beta-adrenoceptors because of its structural resemblance to classical beta-adrenergic agonists [11]. It stimulates the secretion of epinephrine and norepinephrine, which enhances tobacco-driven tumor development [12,13]. Moreover, beta-adrenoceptor antagonists reverse the cancer-promoting action of NNK in human cells derived from colon cancer [14].

In the present study, we investigated the role of NNK in ESCC *in vitro* and *in vivo*, and demonstrated many oncogenic effects of NNK in ESCC, including inhibition of ESCC cell apoptosis as well as promotion of ESCC cell proliferation *in vitro* and tumor growth *in vivo*. We also found that NNK promoted ESCC cell migration and invasion, indicating the potential involvement of NNK in the promotion of tumor metastasis. Moreover, NNK promoted proliferation and inhibited apoptosis of immortalized esophageal epithelial cells. This is consistent with previous study. It has been reported NNK treatment in HET-1A cells could enhance cell proliferation and inhibit cell apoptosis in a dose-dependent manner [21]. In addition, studies have shown chronic exposure of lung alveolar epithelial type II cells to NNK results in malignant transformation and combined NNK and B[a]P enhanced breast cell carcinogenesis chronically induced by PhIP in both non-cancerous and cancerous breast cells[22,23]. These results indicate NNK might promote the malignant transformation of esophageal epithelial cells and play a carcinogenic effect in ESCC. That is, NNK is involved in the formation and development of NNK-related ESCC.

According to our research, knockdown of beta1- and beta2-adrenoceptors inhibited the proliferation, migration and invasion of ESCC cells and induced the apoptosis of both ESCC cells and immortalized esophageal epithelial cells. Consistent with our results, it has been reported stimulation of saltbutamol, a specific beta2-adrenoceptor agonist, promoted the proliferation of human bronchial epithelial cells *in vitro*, and this facilitating effect could be greatly inhibited by treatment with propranolol, a non specific beta-adrenoceptor antagonist[24]. Moreover, we observed the effect of NNK was inhibited to some extent by down-regulation of beta1- and beta2-adrenoceptors with beta2-adrenoceptor showing a more significant effect. In line with our results, beta-adrenoceptor antagonists remarkably inhibit NNK-induced proliferation of HT-29 colon cancer cells, and a beta2-adrenoceptor antagonist is more potent than a beta1-adrenoceptor antagonist [14]. This finding indicates that the beta2-adrenoceptor might play a dominant role in NNK-related ESCC.

As we know, beta-antagonists are commonly and safely used to control arrhythmia and hypertension in patients with cardiovascular diseases for decades. Recently, emerging evidence shows beta- antagonists have additional potential to alleviate the deleterious progression of cancers influenced by beta-adrenergic system [17]. A meta-analysis provides evidence that beta-blocker use can be associated with the prolonged survival of cancer patients, especially patients with early-stage cancer treated primarily with surgery [25]. Another cohort study involved 217,910 patients with hypertensive disease followed up for five years suggested that 9500 patients (4.4%) died from cancer after their first-ever antihypertensive prescription. The proportion of patients who died from cancer was the highest in the calcium channel blocker group (6.5%), followed by thiazide diuretics (4.4%), angiotensin converting enzyme inhibitors (4.2%) and beta-blockers (2.6%) [26]. According to these studies, beta-blocker users had a significant decrease in cancer incidence and mortality compared with non-users. However, contradicting findings were pointed out by other researchers that beta-blockers had no evident beneficial effect on overall survival of patients with cancers in the lung, breast and colon, prostate and pancreatic cancers [27–29]. Thus, further studies are needed to reveal its value in cancer prevention and treatment.

There have been some reports on the mechanism of beta-adrenoceptor-associated carcinogenesis. Results from a study on pancreatic cancer cells suggest that beta-adrenoceptor agonists increase cell proliferation and tumor growth through phosphorylation of ERK1/2 [30]. The ERK pathway phosphorylates Bad, which promotes Bcl-2 to form homodimers to bind Bax and inhibit its activation. This molecular cascade results in proliferation-promoting and anti-apoptotic responses [31]. Furthermore, it has been reported that treatment with beta-adrenergic antagonists significantly suppresses the expression of p-ERK, Akt, Bcl-2, and cyclin D1, and induces the activation of caspase-3, caspase-9, and Bax in cultured pancreatic cancer cells [19]. In line with these studies, our results showed that down-regulation of beta-adrenoceptors significantly decreased the expression of p-ERK1/2, cyclin D1, and Bcl-2, and increased the expression of Bax. NNK promoted the expression of p-ERK1/2, cyclin D1, and Bcl-2, and decreased the expression of Bax. More importantly, the effects of NNK were reversed by down-regulation of beta-adrenoceptors. The immortalized esophageal epithelial cells have the similar activated signaling as ESCC cells. In line with our results, studies have shown NNK treatment alone induced both preneoplastic and neoplastic lesions in the lungs of ferrets, and the expression of p-ERK1/2 and cyclin D1was increased in the lungs of ferrets exposed to NNK [32]. Furthermore, NNK stimulated normal human bronchial cell proliferation through up-regulation of cyclin D1 expression and ERK1/2 was involved in this process, this was confirmed in *vivo* [33]. Therefore, beta-adrenoceptors might mediate the cancer-promoting effect of NNK via regulation of various proteins involved in the pathogenesis of ESCC, which needs to be studied further.

It has been demonstrated that NNK and beta-adrenoceptors are involved in tumor angiogenesis and aggressive growth [17]. A study revealed that norepinephrine induces VEGF expression in several cancer cell lines and the beta-blocker propranolol completely abolishes VEGF production initiated by norepinephrine in cancer cells [34]. Therefore, we detected the expression of VEGF in cells by western blotting, and found that down-regulation of beta-adrenoceptors significantly decreased the expression of VEGF, whereas NNK promoted VEGF expression. More importantly, this effect was reversed by down-regulation of beta-adrenoceptors. Therefore, we further explored the expression of VEGF and CD31 by immunohistochemistry in animal experiments to delineate the role of NNK and beta-adrenoceptors *in vivo*. Our results showed that NNK significantly promoted VEGF and CD31 expression, and increased the MVD, which were reversed by treatment with beta-blockers. These results suggested that NNK promoted tumor growth by inducing the formation of tumor vessels via the beta-adrenoceptor signaling pathway. However, there was no significant difference in VEGF and CD31 expression regardless of treatment with beta-adrenoceptor antagonists, which is not consistent with the previous study. This discrepancy might be associated with differences in genetic backgrounds.

In summary, NNK plays an oncogenetic role through beta-adrenoceptors in ESCC *in vitro* and *in vivo*, and beta2-adrenoceptor plays a more important effect in this process. This novel finding not only reveals a link between NNK and beta-adrenoceptors, but also sheds light on the purported chemoprevention and therapeutic use of beta-adrenoceptor antagonists for the treatment of cigarette smoke-related ESCC.

## Materials and Methods

### Reagents and drugs

Atenolol, ICI118551, NNK, and an anti-beta-actin antibody were purchased from Sigma (St. Louis, MO, USA). Antibodies against beta1- and beta2-adrenoceptors, cyclin D1, VEGF, and CD31 were purchased from Abcam (Cambridge, MA, USA). Antibodies against p-ERK1/2, Bcl-2, and Bax were purchased from Cell Signaling (Beverley, MA, USA). siRNAs targeting beta1- or beta2-adrenoceptor mRNAs and nonspecific negative control siRNA were obtained from Gene Pharma (Shanghai, China).

### Cell culture and treatments

The human ESCC cell lines KYSE410 and KYSE70 was obtained from the Chinese Academy of Medical Sciences and cultured in RPMI 1640 medium (Life Technologies, Carlsbad, CA, USA) supplemented with 10% fetal bovine serum (Life Technologies). The immortalized esophageal epithelial cell line HET-1A was purchased from the American Type Culture Collection (Manassas, VA, USA) and cultured in bronchial epithelium basal medium with growth supplements (Clonetics, San Diego, CA, USA).

Cells were plated at a density of 4×10^5^ cells/well in 6-well plates. After 24 h of culture, the cells were treated for various purposes. To investigate the effect of beta-adrenoceptors, the cells were transfected with siRNA using Lipofectamine 2000 (Life Technologies) according to the manufacturer’s protocol [35]. siRNA sequences were as follows: beta1-adrenoceptor, 5′-CGCUCACCAACCUCUUCAUTT-3′ and 5′-AUGAAGAGGUUGGUGAGCGTT-3′; beta2-adrenoceptor, 5′-GCCUAGCGAUAACAUUGAUTT-3′and 5′-AUCAAUGUUAUCGCUAGGCTT-3′; negative control, 5′-UUCUCCGAACGUGUCACGUTT-3′ and 5′-ACGUGACACGUUCGGAGAATT-3′. To examine the effects of NNK, 0.1 μM NNK was added to the medium directly or at 6 h post-transfection according to our previous study (unpublished data). Cells were collected for further experimentation at 72 h post-treatment as described below.

### RT-PCR

Total RNA was extracted using TRIzol reagent (Life Technologies). Equal amounts of RNA (2 μg) were used to generate first-strand cDNA using Superscript II reverse transcriptase according to the manufacturer’s instructions (Life Technologies). Glyceraldehyde-3-phosphate dehydrogenase (GAPDH) was used as an internal control. The PCR primers were as follows: beta1-adrenoceptor, 5′-GACGCTCACCAACCTCTTCA-3′ and 5′-CACAGCTCGCAGAAGAAGGA-3′; beta2-adrenoceptor, 5′-CATTCTGATGGTGTGGATTGTGTC-3′ and 5′-CAGCAGGTCTCATTGGCATAGC-3′; GAPDH, 5′-GGTGGTCTCCTCTGACTTCAACA-3′ and 5′-GTTGCTGTAGCCAAATTCGTTGT-3′. Cycling conditions were as follows: 94°C for 5 min, 35 cycles of 94°C for 20 s, a specific annealing temperature for each gene for 20 s, and 72°C for 30 s, followed by a final extension of 7 min at 72°C.

### Western blot analysis

Cells were lysed with radioimmunoprecipitation buffer containing 1 mM phenylmethylsulfonyl fluoride, 10 μg/ml aprotinin, and 10 μg/ml leupeptin as described previously [20]. Thirty micrograms of total protein were fractionated by SDS-polyacrylamide gel electrophoresis and then transferred to nitrocellulose membranes (Amersham, Arlington Heights, Illinois, USA). The membranes were probed with primary antibodies overnight at 4°C and then incubated for 1 h with secondary peroxidase-conjugated antibodies, followed by detection with an enhanced chemiluminescence system (Pierce, Rockford, IL, USA). Beta-actin was used as the loading control.

### Cell proliferation assay

To evaluate the effects of NNK and beta-adrenoceptors on the proliferation of KYSE410 and HET-1A cells, we used a MTT assay [18]. Briefly, 2×10^4^ cells of the variously treated groups were seeded in 96-well plates. After 48 h, the medium was removed and MTT (5 mg/ml; Life Technologies) was added to each well. Dimethyl sulfoxide (DMSO) was then added and the OD of each well was read at 490 nm. Three independent experiments were performed in triplicate.

### Detection of apoptosis by flow cytometry

Apoptosis was measured using an Annexin V-FITC/PI Kit (Becton Dickinson, Franklin Lakes, NJ, USA) following the manufacturer's instructions. Briefly, cells were collected, washed with cold phosphate-buffered saline (PBS), and then resuspended in the Binding Buffer at a final concentration of 1×10^6^ cells/ml. One hundred thousand cells were transferred to FACS tubes and incubated with 5 μl annexin V-FITC and 5 μl PI for 20 min at 25°C in the dark. Four hundred microliters of Binding Buffer was then added to each tube followed by immediate flow cytometry (LSRFortessa, Becton Dickinson). A minimum of three independent experiments were performed.

### *In vitro* migration and invasion assays

Cancer cell migration and invasion assays were performed according to our previous study [20] with some modification. BD chambers (Becton Dickinson) containing a polyethylene terephthalate membrane filter (6.4 mm diameter; 8 μm pore size) were used for the migration assay and BD BioCoat^™^ Matrigel^™^ Invasion Chambers (Becton Dickinson) containing a polyethylene terephthalate membrane filter (6.4 mm diameter; 8 μm pore size) precoated with a thin layer of matrigel was used for the invasion assay. Briefly, the upper chamber was seeded with 1×10^5^ cells suspended in 0.5 ml serum-free medium. The lower chamber contained 0.75 ml complete medium. After 24 h of culture, non-migratory or non-invasive cells on the upper surface of the membrane were wiped off. Migratory or invasive cells on the bottom surface were fixed with methanol, stained with hematoxylin and eosin, and then counted under a microscope.

### Ethics statement

All animal studies were approved by the Institutional Animal Care and Use Committee of Capital Medical University. All surgical procedures were performed under isoflurane anesthesia, and all efforts were made to minimize suffering.

### Cell implantation

KYSE410 cells were dissociated, collected, and resuspended in PBS at a density of 5×10^6^ cells/ml. Thirty-six 5-week-old male athymic nude mice (BALB/c nu/nu; 16–18 g) were purchased from the Institute of Laboratory Animal Science, Peking Union Medical College. Each mouse was subcutaneously injected with KYSE410 cells (1×10^6^ cells in 0.2 ml PBS). After 1 week, the mice were randomized into six groups with six animals in each group to receive various treatments: DMSO (vehicle), atenolol (10 mg/kg/day), ICI118551 (10 mg/kg/day), NNK (1.2 mg/kg/day), atenolol+NNK, and ICI118551+NNK. They were lavaged with DMSO/NNK every day and intraperitoneally injected with DMSO/atenolol/ICI118551 three times per week for 4 weeks. Body weight and tumor diameters were measured at regular intervals with digital calipers. The tumor volume was calculated using the formula: volume (mm^3^) = (width)^2^ × length/2. Animals were sacrificed at 5 weeks post-implantation. Tumors were dissected, weighted, and fixed in 10% neutral buffered formalin for further analysis.

### Immunohistochemistry

The distribution of VEGF and CD31 in ESCC xenografts was determined by immunohistochemistry as described previously [20]. Fixed tissues were embedded in paraffin for sectioning. Five micrometer-thick paraffin sections were deparaffinized and heated for antigen retrieval in sodium citrate buffer. Then, endogenous peroxidases were inactivated with 3% hydrogen peroxide and non-specific binding sites were blocked with 10% normal goat serum for 1 h. The sections were then incubated with the following primary antibodies: mouse monoclonal anti-VEGF (1:100) or anti-CD31 (1:100) at 4°C overnight, followed by a horseradish peroxidase conjugated secondary antibody for 1 h. Finally, the sections were washed, developed with diaminobenzidine, counterstained with hematoxylin, dehydrated, and then mounted with DPX.

VEGF intensity was assessed on a four-point scale as follows: 0, negative; 1, weak staining; 2, intermediate staining; 3, strong staining. The percentage of stained cells was assessed on a six point scale as follows: 1, 0–4%; 2, 5–19%; 3, 20–39%; 4, 40–59%; 5, 60–79%; 6, 80–100%. The scores for intensity and proportion were then multiplied for a composite score from 0 to 18 according to the method of Detre *et al* [36].

MVD was assessed using the method of Weidner *et al* [37]. The distribution of blood vessels was first observed at low magnification (40 or 100×), and then five regions were selected where the vascular was the most dense. At high magnification (400×), the number of blood vessels was counted in each field. The average value of the selected five fields was considered to be the number of microvessels in each specimen. The microvascular criteria were: brown staining, vessels clear and separated from the surrounding tumor cells and connective tissue, and endothelial cells or cell clusters considered as a single microvessel. Lumens were not counted when the diameter was larger than 50 μm with a thick muscle layer.

### Statistical analysis

All statistical analyses were performed using SPSS version 12.0 (SPSS, Chicago, IL, USA). The results are presented as the mean ± SD. Differences between groups were assessed by analysis of variance (ANOVA) or Student’s t-test. A value of *p*<0.05 was considered statistically significant.

## Supporting Information

S1 Fig Expression of beta1- and beta2-adrenoceptors of KYSE70 cells was effectively knocked down using siRNA. siRNA-induced down-regulation of beta1- and beta2-adrenoceptor mRNA and protein expression of KYSE70 cells was confirmed by RT-PCR (A) and western blotting (B), respectively. GAPDH and beta-actin served as endogenous controls. Negative is representative of nonspecific control siRNA for beta-adrenoceptors.
(TIF)

S2 Fig Effect of NNK and beta-adrenoceptors on cell proliferation of KYSE70 cells. Variously treated KYSE70 cells were seeded in triplicate into 96-well plates. At the indicated time point, the medium was removed, MTT and DMSO were added sequentially, and the ODs were measured using a microplate reader. Data were obtained from three independent experiments. ^ε^ p<0.05 compared with the blank group; ^ζ^ p<0.05 compared with the negative group; ^ξ^ p<0.05 compared with the negative+NNK group. Negative is representative of nonspecific control siRNA for beta-adrenoceptors.
(TIF)
