# Supplementary figures and images for: 4-(Methylnitrosamino)-1-(3-Pyridyl)-1-Butanone Promotes Esophageal Squamous Cell Carcinoma Growth via Beta-Adrenoceptors In Vitro and In Vivo
Source: PLoS One. 2015 Mar 5;10(3):e0118845. doi: 10.1371/journal.pone.0118845 (PMC4351054; doi:10.1371/journal.pone.0118845)

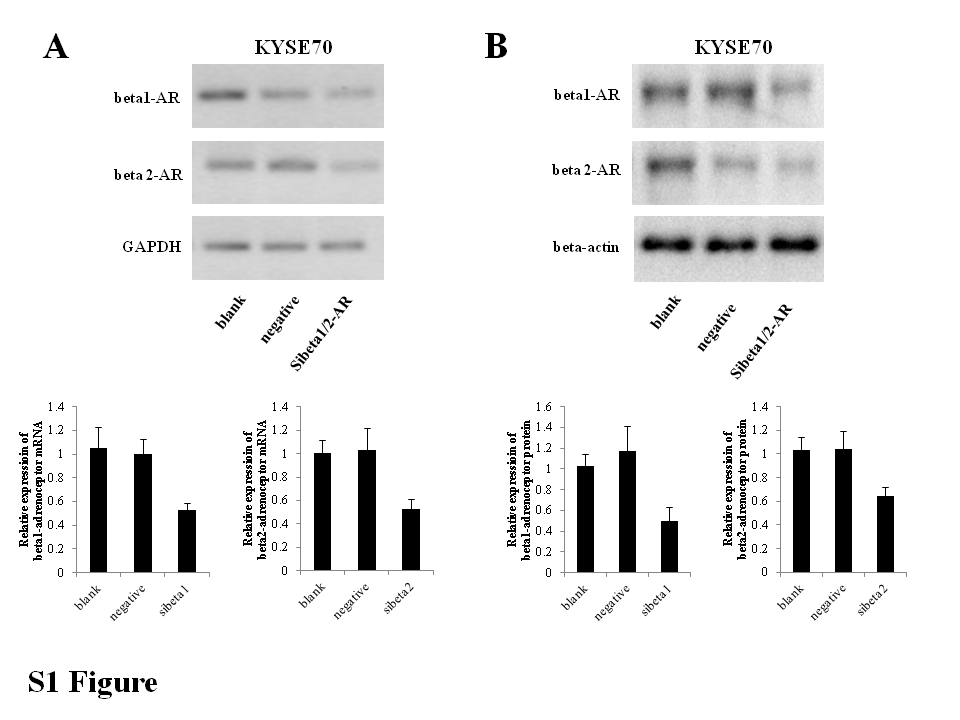

Supplement: S1 Fig — siRNA-induced down-regulation of beta1- and beta2-adrenoceptor mRNA and protein expression of KYSE70 cells was confirmed by RT-PCR (A) and western blotting (B), respectively. GAPDH and beta-actin served as endogenous controls. Negative is representative of nonspecific control siRNA for beta-adrenoceptors. (TIF) [file pone.0118845.s001.tif]

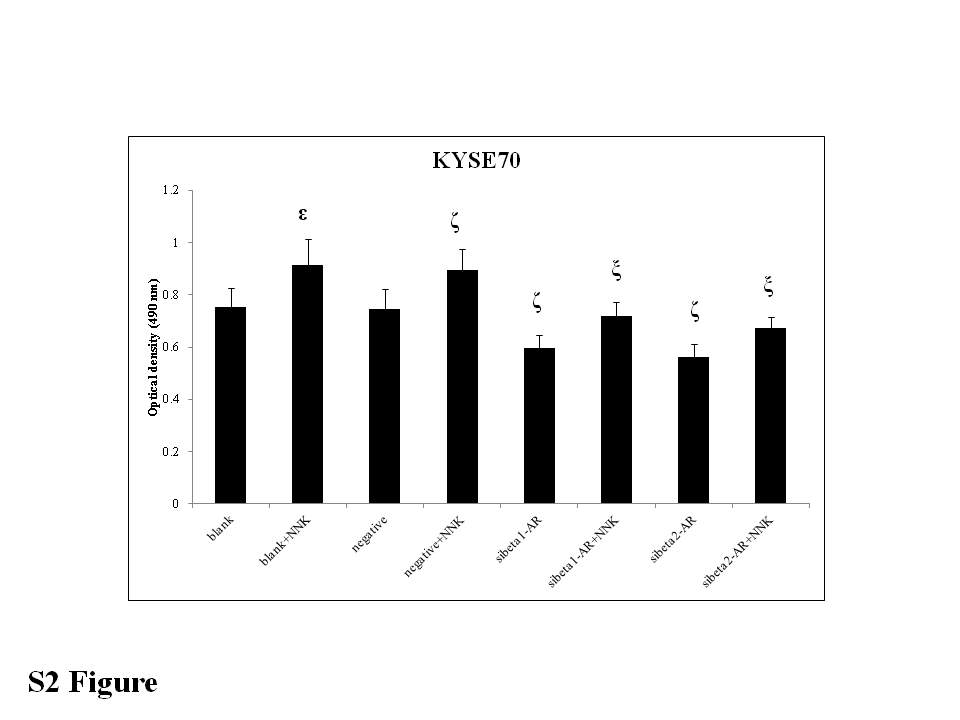

Supplement: S2 Fig — Variously treated KYSE70 cells were seeded in triplicate into 96-well plates. At the indicated time point, the medium was removed, MTT and DMSO were added sequentially, and the ODs were measured using a microplate reader. Data were obtained from three independent experiments. ε p<0.05 compared with the blank group; ζ p<0.05 compared with the negative group; ξ p<0.05 compared with the negative+NNK group. Negative is representative of nonspecific control siRNA for beta-adrenoceptors. (TIF) [file pone.0118845.s002.tif]
